# Retrospective analysis of the relationship between bone mineral density and body composition in a health check-up Chinese population

**DOI:** 10.3389/fendo.2022.965758

**Published:** 2022-08-10

**Authors:** Yuxin Li, Zhen Huang, Yan Gong, Yansong Zheng, Qiang Zeng

**Affiliations:** ^1^ Second Medical Center and National Clinical Research Center for Geriatric Diseases, Chinese People’s Liberation Army General Hospital, Beijing, China; ^2^ Academy of Medical Engineering and Translational Medicine, Tianjin University, Tianjin, China; ^3^ Nanning First People’s Hospital (The Fifth Affiliated Hospital of Guangxi Medical University), Nanning, China

**Keywords:** physical examination, body weight, muscle mass, skeletal muscle mass index, bone mineral density, osteoporosis

## Abstract

**Purpose:**

This study was designed to explore the relationship between bone mineral density (BMD) and body composition indicators in Chinese adults (≥50 years) in order to provide a scientific basis for optimal bone health management.

**Method:**

Individuals ≥50 years old who received physical examinations and routine check-ups at the Health Management Research Institute of PLA General Hospital from September 2014 through March 2022 were included as research subjects in this study. Basic clinical and demographic information were recorded for all subjects, along with smoking and drinking status, height and body weight. A panel of routine blood chemistry and metabolite markers were measured, along with lean muscle mass and body fat mass using body composition bioelectrical impedance analysis (BIA). Body mass index (BMI), body fat percentage (BFP), skeletal muscle mass index (SMI), and bone mineral density (BMD) were calculated for all individuals. For comparative analysis, individuals were grouped based on their BMI, BFP, SMI and BMD T-score. Follow-up examinations were performed in a cohort of 1,608 individuals matched for age, sex, smoking and drinking history for ≥5 years,

**Results:**

In this large cross-sectional study, age, smoking, homocysteine (Hcy) and blood glucose levels were established as independent risk factors for osteoporosis. Multi-factor logistic regression analysis showed that age, sex, BMI, intact parathyroid hormone (iPTH), SMI, BFP, smoking, blood levels of inorganic phosphate (P) and K+ were all significantly associated with osteoporosis risk (P<0.05). A subset of these factors- BMI, SMI, BFP and K+, were determined to be protective. In the cohort followed for ≥5 years, SMI and BMD decreased while BFP and BMI increased significantly (P<0.001) over time.

**Conclusion:**

Risk of osteoporosis may be reduced by increasing body weight, particularly lean muscle mass, while simultaneously controlling BFP.

## Introduction

Osteoporosis is a common musculoskeletal disorder among the elderly and a chronic condition which, like many other chronic conditions, requires long-term clinical management (1). This disorder frequently leads to fragility, bone fractures, chronic pain and other symptoms, culminating in a reduced quality of life, disability and death. From 2005 to 2013, the disability-adjusted life year (DALY) for the global population as a result of musculoskeletal disorders increased by 17.7% (2). Another study reported that from 2008 to 2018, 45.9% of Chinese women aged ≥ 65 years suffered from osteoporosis of the lumbar vertebra, hip or femur, while the incidence of osteoporosis for men and women individually aged ≥ 60 years was 6.46% and 29.13%, respectively (3). In 2010, the total number of individuals aged ≥ 50, the age group at highest risk of osteoporotic fractures, reached 158 million. That number is expected to double by 2040 (4, 5). Thus, early screening and intervention for osteoporosis have become important clinical tactics for keeping rates of related fractures and morbidities to the lowest levels possible in this population.

Bone mineral density (BMD) is defined as the mass of bone mineral per unit volume. It is considered the gold-standard indicator of skeletal metabolic status, and used for analyzing the change of bone mass over time. The T-score, which refers to the number of standard deviations that an individual’s BMD differs from the peak bone mass of a young healthy individual of the same sex, is the most meaningful indicator for osteoporosis in men aged ≥ 50, and in post-menopausal women. BMD is associated with a variety of factors such as age (6), weight (7), nutrition (8), exposure to sunlight, premature menopause (9), smoking, drinking, genetic factors (10), sex (11, 12), and exercise. Among these factors, heredity, sex and age are unmodifiable, while weight, nutrition, exercise, exposure to sunlight, and lifestyle are modifiable. Body composition indicators such as BMI, BFP and skeletal muscle mass index (SMI) (13) are a result of the combined effects of unmodifiable and modifiable factors on the human body. Therefore, the ultimate impact of these factors can be reflected by body composition indicators. SMI, the percentage of skeletal muscle mass out of total body weight, is a widely recognized indicator used to assess skeletal muscle health and even help diagnose sarcopenia (14, 15).

The interrelationship of body composition and osteoporosis is complex and multifactorial. Possibly because of differences in ethnicity, nutrition, lifestyle habits, and body size or even algorithms, the conclusions of the current available correlation studies are conflicting. At the same time, the American or European guidelines may not applicable to Asians. The related Chinese population has been less studied. A study completed a 3-year follow-up of 208 men from the Foshan community in Guangdong, China, and this prospective study concluded that bone density at sites other than the skull throughout the body was positively correlated with human skeletal muscle mass parameters, especially SMI, however, the sample size was small, and the follow-up period was only 3 years (16).

Body composition is dictated not only by unmodifiable factors such as heredity, sex and aging (17), but also by acquired lifestyle factors which are very modifiable. Indeed, BMI, BFP and SMI can be modified through a variety of weight control tactics, particularly exercise (14, 18). Body composition can thus be viewed as an aggregate outcome of the cumulative effect of unmodifiable and modifiable factors in the human body. Body composition indicators, therefore, may be useful not only as early predictors of BMD risk, but also as indicators of BMD intervention effectiveness.

Health check-up belongs to opportunistic screening. Although this kind of screening has certain limitations, with the popularization of physical examination in China, the practical significance of this kind of screening is very noteworthy. It is not necessary to make an accurate diagnosis. Finding the tendency of osteoporosis in advance and urging people to intervene in advance can produce good results (19). Because osteoporosis often shows no clinical symptoms in early stages, this condition can only be diagnosed through a combination of objective and sometimes subjective clinical tests. However, previous studies have shown that quantifiable data acquired through peripheral dual-energy X-ray absorptiometry can reveal trends of BMD (20, 21).

This study examined the relationship between BMD and body composition markers, especially SMI, in individuals aged ≥ 50, with the objective of providing data to support clinicians tasked with counseling patients on osteoporosis prevention.

## Methods

### Study population

**Figure 1:**
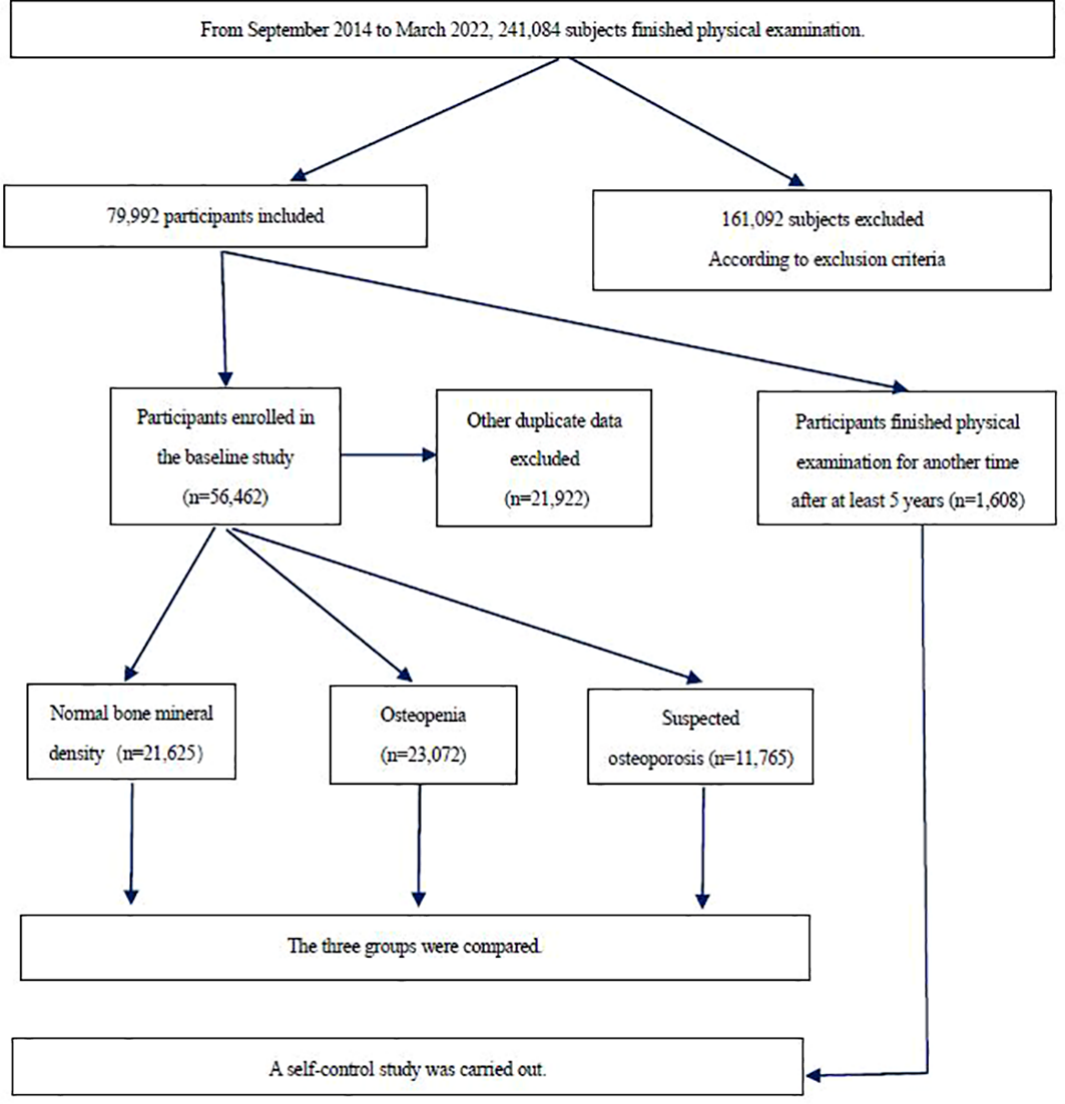
Flow chart of the study population.

All individuals (aged ≥ 50) who received physical examinations and completed related checks at the Health Management Research Institute of PLA General Hospital from September 2014 through March 2022 were included in this study. A total of 56,462 individuals were included in the baseline study- 32,510 males (57.58%) and 23,952 females (42.42%). Average age of this cohort was 55.95 ± 5.40 years. A subset of 1,608 individuals completed a follow-up examination ≥ 5 years after the initial check. Of these, 1,097 were male (68.22%) and 511 female (31.78%). Exclusion criteria included pre-menopausal women, patients with severe cardiac or renal insufficiency, limb differences or mobility impairments, patients with confirmed malignancies, primary hyper-parathyroidism or Cushing’s syndrome, post-gastrectomy, and patients with prescriptions for corticosteroids (22). For individuals who received more than one physical examination during the study period, only results of their first physical examination were taken as baseline data for analysis. See **Figure 1** for details of the selection process. The retrospective study protocol was approved (S2019-190-02) by the Chinese People’s Liberation Army General Hospital ethics committee. All individuals enrolled were informed that their physical examination data would be de-identified, and signed consent documents.

### Lifestyle survey

We input the lifestyle questionnaire into the computer in advance, so that the subjects can input lifestyle information by themselves during the physical examination by means of touch-screen input. Data were collected concerning each subject’s basic demographic information, smoking and drinking habits. Smoking was defined as smoking ≥ 10 cigarettes per day for ≥ 1 year, according to the standards in the relevant literature (23), and those who fail to meet the standards are defined as non-smoking. Anyone who smokes less than one cigarette a day and can maintain it for more than a year is called quitting smoking, according to the Chinese clinical smoking cessation guidelines (2015 Edition) (24). Drinking included limited drinking (no drinking, or drinking ≤ 25g of alcohol/day for a male adult, and ≤ 15g of alcohol/day for female adult). Excessive drinking refers to drinking ≥ 25g of alcohol/day for males, and ≥ 15g alcohol/day for females) (25).

### Physical examination and body composition measurement

Subjects’ height, weight, blood pressure and other vital physiological parameters were captured during routine examination. Weight is measured by electronic scale, and height is measured by Infrared height measuring instrument (OMRON, HNH-318, Japan). All these indicators are obtained according to the quality control standards of physical examination. A body composition analyzer (Inbody720, South Korea) was used to measure body composition indicators. For these measurements, an individual in resting state would stand barefoot on the analyzer, arms hanging down in relaxed state, with the front of the soles, heels, thumbs and palms in contact with eight different electrodes. Bioelectrical impedance values would then be measured to obtain body fat and muscle mass, allowing for calculation of other indicators. Height and weight were used to calculate BMI, BFP (body fat mass/weight x 100%) and SMI (muscle mass/weight x 100%). Group classification based on BMI included- underweight (<18.5 kg/m^2^), normal weight (18.5-23.9 kg/m^2^), overweight (24.0-27.9 kg/m^2^), and obese (≥28.0 kg/m^2^) (26). Test results of SMI were arranged into three levels in ascending order- low, moderate and high- divided at 25% and 75%, forming the three groups of low SMI, moderate SMI, and high SMI. Similarly, test results of BFP were arranged, into three levels in ascending order- low, moderate and high- also divided at 25% and 75%, forming the three groups of low BFP, moderate BFP, and high BFP.

### Biochemical Parameters

Venous blood was collected from all subjects following an overnight fast, and according to the quality control and testing standards of the Clinical Laboratory of PLA General Hospital (27). Levels of total cholesterol (TC), triglyceride (TG), high density lipoprotein cholesterol (HDL-C), low density lipoprotein cholesterol (LDL-C), fasting blood glucose (FBG), hemoglobin A1c (HbA1c), Ca^2+^, K^+^ and inorganic phosphorus (P), as well as intact parathyroid hormone (iPTH) and total 25-hydroxy-vitamid D (25 (OH)D) were measured in serum samples using electrochemiluminescence method; the enzymatic cycling method was adopted for the measurement of homocysteine (Hcy) (28).

### BMD measurement

A dual-energy x-ray bone density device (Osteosys EXA 3000 (GSYJX (J) 2009 No. 3312468), South Korea) was used for the measurement of BMD at one-third distal radius to obtain the mean forearm BMD and T-score. Diagnosis of osteoporosis was determined based on WHO-recommended standards of 1994: Normal BMD = T-score ≥ 1.0 SD; osteopenia = −2.5 SD < T-score < −1.0 SD; suspected osteoporosis = T-score ≤ −2.5 SD (29).

### Follow-up examinations

Individuals included in the baseline study were considered to have completed a follow-up examination if they received an examination ≥ 5 years after their initial visit and examination. A longitudinal analysis was then performed over time for each of these subjects.

### Statistical analysis

Coded and quantified questionnaire data were analyzed using Stata 11.0. Body composition and blood marker data were expressed as mean ± SD and categorical data were expressed as percentages. For group comparisons, the χ^2^ test, *t*-test, and one-way analysis of variance were carried out. Pairwise comparisons were made using Bonferroni method and multivariate analysis by using logistic regression analysis. For every comparison, *P*<0.05 indicated a statistically significant difference.

## Results

### Results of baseline BMD screening

A total of 23,072 individuals (40.86%) were determined to have normal BMD, 21,625 (38.30%) had osteopenia, and 11,765 (20.84%) were suspected to have osteoporosis. The mean BMD in the overall cohort was 0.453 ± 0.099g/cm^2^. Summaries of blood markers and smoking/drinking in the overall cohort are shown in **Table 1**.

**Table 1:** Comparison of three groups of basic data and clinical indexes (n=56,462).

	Normal BMD (n=21,625)	Osteopenia (n=23,072)	Suspected osteoporosis (n=11,765)	Statistics
**Gender**				χ2 = 357.43, *P*<0.001
Male	13,063 (60.41)	13,559 (58.77)	5,888 (50.05)	
Female	8,562 (39.59)	9,513 (41.23)	5,877 (49.95)	
**Smoking status**				χ2 = 25.81, *P*<0.001
Non-smoking	14,688 (67.92)	15,303 (66.33)	7,868 (66.88)	
Quit smoking	1,657 (7.66)	1,834 (7.95)	820 (6.97)	
Smoking	5,280 (24.42)	5,935 (25.72)	3,077 (26.15)	
**Drinking status**				χ2 = 110.17, *P*<0.001
Never drinking or small amount of alcohol	15,190 (70.24)	16,320 (70.74)	8,868 (75.38)	
Excessive drinking	6,435 (29.76)	6,752 (29.26)	2,897 (24.62)	
**Age(year)**	54.19 ± 4.39	55.87 ± 5.07*	59.33 ± 6.09*#	F=3942.38, *P*<0.001
**BFP (%)**	26.08 ± 5.70	26.78 ± 5.62*	27.59 ± 5.92*#	F=273.25, *P*<0.001
**BMI (kg/m2)**	25.18 ± 3.09	25.15 ± 3.24	24.89 ± 3.44*#	F=35.59, *P*<0.001
**SMI(%)**	68.33 ± 5.64	67.62 ± 5.53*	66.81 ± 5.81*#	F= 282.64, *P*<0.001
**BMD(g/cm2)**	0.535 ± 0.069	0.437 ± 0.058*	0.332 ± 0.071*#	F=38091.13, *P*<0.001
**TC (mmol/L)**	4.81 ± 0.92	4.89 ± 0.95*	4.93 ± 0.97*#	F=75.07, *P*<0.001
**TG (mmol/L)**	1.69 ± 1.25	1.73 ± 1.25*	1.68 ± 1.19#	F=7.04, *P*=0.0009
**LDL-c (mmol/L)**	3.10 ± 0.81	3.16 ± 0.84*	3.19 ± 0.86*#	F=46.72, *P*<0.001
**HDL-C (mmol/L)**	1.29 ± 0.33	1.31 ± 0.34*	1.34 ± 0.35*#	F=65.41, *P*<0.001
**FBG (mmol/L)**	5.93 ± 1.46	6.00 ± 1.51*	6.09 ± 1.61*#	F=44.54, *P*<0.001
**HbA1c (%)**	6.01 ± 0.88	6.07 ± 0.89*	6.17 ± 0.97*#	F=122.69, *P*<0.001
**Hcy(μmol/L)**	12.56 ± 6.31	12.78 ± 6.33*	3.20 ± 6.49*#	F=37.40, *P*<0.001
**Ca^2+^ (mmol/L)**	2.332 ± 0.086	2.337 ± 0.083*	2.337 ± 0.086*	F=20.18, *P*<0.001
**K^+^ (mmol/L)**	4.256 ± 0.307	4.249 ± 0.309*	4.239 ± 0.321*#	F=12.28, *P*<0.001
**P (mmol/L)**	1.163 ± 0.155	1.176 ± 0.151*	1.188 ± 0.149*#	F=111.74, *P*<0.001
**iPTH (pg/ml)**	45.35 ± 15.71 (n=2,149)	6.50 ± 16.45 (n=2,526)	48.91 ± 21.05*#(n=1,302)	F=17.25, *P*<0.001
**25(OH)D (nmol/L)**	17.97 ± 7.64 (n=2,149)	17.91 ± 7.75 (n=2,526)	18.22 ± 7.84(n=1,302)	F=0.72, *P=0.4884*

BMI, body mass index; BMD, forearm bone mineral density; BFP, body fat percent (body fat/weightx100%); SMI, skeletal muscle mass index (total muscular mass/weight x100%); TC, Total cholesterol; TG, Triglyceride; HDL-C, high density lipoprotein cholesterol; LDL-C, low density lipoprotein cholesterol; FBG, fasting blood glucose; HbA1c, hemoglobin A1c; Hcy, blood homocysteine; P, inorganic phosphorus; iPTH, Intact parathyroid hormone.

*compared with normal bone mineral density, P<0.05.

#compared with osteopenia, P<0.05.

### Compare of blood markers and lifestyle factors associated with BMD

**Figure 2:**
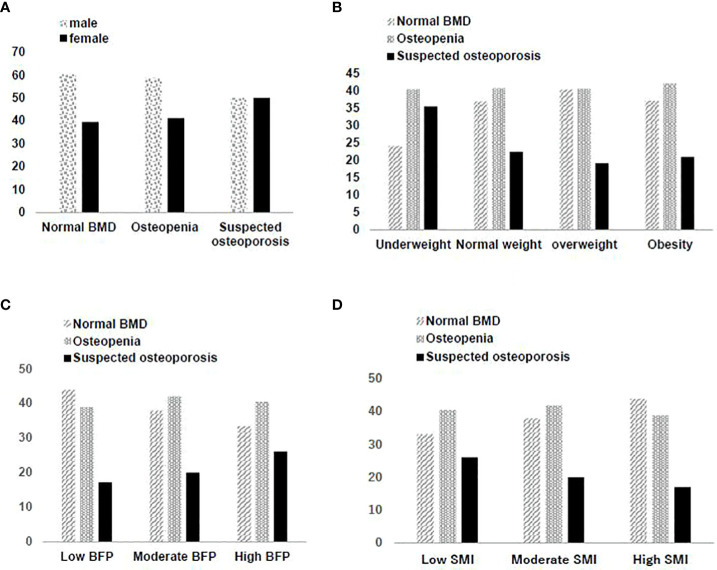
**(A)** Gender distribution in different bone mineral density screening results. **(B)** Distribution of bone mineral density screening results in different BMI groups. **(C)** Distribution of bone mineral density screening results in different BFP groups. **(D)** Distribution of bone mineral density screening results in different SMI groups.

A significant increase was observed in TC, LDL-C, HDL-C, FBG, HbA1c, Hcy, and BFP, in contrast to a decrease in SMI and BMD among the three BMD groups. Compared with the normal group, both the suspected osteoporosis group and the osteopenia group, the latter in particular, had a larger share of individuals who reported limited drinking compared with those who reported excessive drinking. This is contrary to results of most previous studies and can presumably be attributed to a significantly higher percentage of males in the normal group. Despite a higher percentage of males than females in the cohort overall, the percentage of females is lowest in the normal group, higher in the osteopenia group and highest in the suspected osteoporosis group where the percentage of females is almost the same as that of males. See **Figure 2A**.

### Multivariate analysis of osteoporosis screening results

**F**or results of multiple logistics regression in which osteoporosis was used as the dependent variable, and other factors as independent variables. The age, sex, BMI, iPTH, SMI, BFP, smoking, P and K+ were all determined to be significantly associated with (*P*<0.001) osteoporosis. Of these, BMI, SMI, BFP and K+ were determined to be protective factors. See in **Table 2**.

**Table 2:** Results of multiple logistic regression analysis **(n=56,462)**.

	Odds Ratio	z	P>|z|	[95% Confidence Interval]
**Age (year)**	1.158	22.49	0.000	1.143, 1.174
**Gender**	1.972	4.53	0.000	1.470, 2.644
**BMI (kg/m2)**	0.885	-6.12	0.000	0.851, 0.920
iPTH	1.009	4.47	0.000	1.004, 1.013
**SMI(%)**	0.728	-4.11	0.000	0.625, 0.847
**BFP (%)**	0.766	-3.65	0.000	0.664, 0.884
**Smoking status**	1.182	3.42	0.001	1.074, 1.301
**P (mmol/L)**	1.908	2.62	0.009	1.176, 3.096
**K^+^ (mmol/L)**	0.750	-2.57	0.010	0.602, 0.934
**Drinking status**	1.071	1.50	0.134	0.979, 1.170
**Hcy(μmol/L)**	1.001	1.27	0.205	0.996, 1.019
**Ca^2+^ (mmol/L)**	0.686	-0.87	0.384	0.293, 1.603
**FBG (mmol/L)**	1.031	0.79	0.427	0.956, 1.111
**TG (mmol/L)**	0.944	-0.80	0.427	0.819, 1.088
**HDL-C (mmol/L)**	0.881	-0.58	0.562	0.574, 1.351
**HbA1c (%)**	1.027	0.39	0.699	0.898, 1.172
**TC (mmol/L)**	1.045	0.23	0.818	0.718, 1.521
**LDL-c (mmol/L)**	1.026	0.13	0.894	0.703, 1.498
**25(OH)D (nmol/L)**	1.000	0.07	0.943	0.992, 1.009

BMI, body mass index; BFP, body fat percent (body fat/weightx100%); SMI, skeletal muscle mass index (total muscular mass/weight x100%); TC, Total cholesterol; TG, Triglyceride; HDL-C, high density lipoprotein cholesterol; LDL-C, low density lipoprotein cholesterol; FBG, fasting blood glucose; HbA1c, hemoglobin A1c; Hcy, blood homocysteine; P, inorganic phosphorus; iPTH, Intact parathyroid hormone.

### Body composition test results

Based on their BMI, 802 individuals (1.42%) were underweight, 19,889 (25.23%) were normal, 25,802 (45.70%) were overweight, and 9,969 (17.66%) were obese. The division points at 25% and 75% of SMI corresponded to 63.88% and 71.36% of the individuals involved, thereby 14,113 individuals were determined to have low SMI, 28,230 had moderate SMI, and 14,119 had high SMI. The division points at 25% and 75% of BFP corresponded to 23.0% and 30.6% of the individuals, thereby 14,316 individuals had low BFP, 27,971 had moderate BFP, and 14,175 had high BFP.

There were significant differences among the three groups (normal, osteopenia, suspected osteoporosis) in BMI (*F*=35.59, *P*<0.001). A pairwise comparison found the normal and the Osteopenia group to have no significant differences in BMI, while the suspected osteoporosis group had the lowest BMI. There was a significant positive correlation (*β*=0.1446, *P*<0.001) between BMD and BMI (**Figure 2B**). The underweight group had the highest share of suspected osteoporosis cases (*χ2 =* 231.57, *P*<0.001).

BFP showed an upward trend (*F*=273.25, *P*<0.001) from the normal group to the suspected osteoporosis group which was determined to be significant with pairwise comparison. There also was a significant negative correlation (*β*=-0.3839, *P*<0.001) between BMD and BFP (**Figure 2C**). The group with a high BFP had the highest rate of suspected osteoporosis (*χ2 =* 524.72, *P*<0.001).

SMI showed a downward trend (*F*=282.64, *P*<0.001) from the normal group to the suspected osteoporosis group which was determined to be significant with pairwise comparison. There was a significant positive correlation (*β*=0.3855, *P*<0.001) between BMD and SMI (**Figure 2D**). The group with low SMI had the highest rate of suspected osteoporosis, and the group with high SMI had the lowest rate of suspected osteoporosis (*χ2 =* 538.85, *P*<0.001).

### Longitudinal analysis

To explore the change of body composition and bone mass over time, follow-up examinations were conducted ≥ 5 years later on 1,608 individuals matched for age, sex, and histories of smoking and drinking. Longitudinal analysis of the endpoints recorded at both examinations were performed. **Table 3** shows summary analysis of differences between baseline data and re-examination in this cohort after ≥ 5 years. Age, BFP, BMI, FBG, HbA1c, Ca^2+^, K^+^ and 25-(OH)D increased from baseline levels, while SMI, BMD, TC and LDL-C decreased.

**Table 3:** Comparison of clinical data before and after completion of follow-up (n=1,608).

	Baseline (n=1,608)	Follow-up (n=1,608)	Mean change	Statistics
**Age (year)**	53.89 ± 3.87	59.42 ± 3.98	5.53	*t*=-39.915, *P*<0.001
**BFP (%)**	24.94 ± 5.44	27.61 ± 5.53	2.68	*t* =-13.831, *P*<0.001
**BMI (kg/m^2^)**	24.68 ± 2.93	25.21 ± 2.99	0.52	*t* =-4.963, *P*<0.001
**SMI (%)**	69.68 ± 5.34	66.97 ± 5.52	-2.71	*t* =14.156, *P*<0.001
**BMD (g/cm^2^)**	0.478 ± 0.089	0.457 ± 0.097	-0.021	*t* =6.316, *P*<0.001
**TC (mmol/L)**	4.86 ± 0.89	4.69 ± 0.94	-0.17	*t* = 5.287, *P*<0.001
**TG (mmol/L)**	1.68 ± 1.10	1.66 ± 1.10	-0.02	*t* =0.487, *P*= 0.626
**LDL-c (mmol/L)**	3.14 ± 0.79	3.03 ± 0.84	-0.11	*t* =3.824, *P=*0.001
**HDL-C (mmol/L)**	1.30 ± 0.34	1.29 ± 0.34	-0.008	*t* =0.739, P=0.460
**FBG (mmol/L)**	5.79 ± 1.18	5.95 ± 1.30	0.15	*t* =3.378, *P=0.001*
**HbA1c (%)**	5.90 ± 0.74	6.06 ± 0.79	0.16	*t* = -5.845, *P*<0.001
**Hcy (μmol/L)**	12.32 ± 5.99	12.41 ± 5.33	0.09	*t* =0.419, *P=0.675*
**Ca^2+^ (mmol/L)**	2.325 ± 0.085	2.335 ± 0.086	0.010	*t* =3.279, *P=0.001*
**K^+^ (mmol/L)**	4.226 ± 0.292	4.331 ± 0.318	0.105	*t* = 9.541, *P*<0.001
**P (mmol/L)**	1.156 ± 0.152	1.153 ± 0.154	0.003	*t* =0.506, *P=0.613*
**iPTH (pg/ml)**	46.68 ± 14.31	46.49 ± 16.96	0.191	*t* =0.089, *P=0.929*
**25 (OH)D (nmol/L)**	15.38 ± 6.67	19.11 ± 8.42	3.73	*t* =3.5547, *P=0.0004*

BMI, body mass index; BMD, forearm bone mineral density; BFP, body fat percent (body fat/weightx100%); SMI, skeletal muscle mass index (total muscular mass/weight x100%); TC, Total cholesterol; TG, Triglyceride; HDL-C, high density lipoprotein cholesterol; LDL-C, low density lipoprotein cholesterol; FBG, fasting blood glucose; HbA1c, hemoglobin A1c; Hcy, blood homocysteine; P, inorganic phosphorus; iPTH, Intact parathyroid hormone.

Compared with the baseline examination, rates of suspected osteoporosis significantly increased (*χ2 =* 36.8862, *P*<0.001) after ≥ 5 years. A comparison of the association between BMD and BMI, BFP and SMI at baseline and follow-up is shown in **Table 4**. The high SMI group was determined to have the highest BMD at both time points. BMD typically decreases over time, but individuals with a higher SMI have greater bone mass and thus have a lower rate of osteoporosis. Similarly, individuals with a low BFP have the highest BMD.

**Table 4:** The self-control study of BMD grouped by BMI, BFP and SMI. .

		Baseline (n=1,608)						Follow-up (n=1,608)					
	Normal BMD	Osteopenia	Suspected osteoporosis	Total	BMD		Normal BMD	Osteopenia	Suspected osteoporosis	Total			BMD
BMI grouping														
Low weight	2 (9.09)	16 (72.73)	4 (18.18)	22	0.447±0.114		0 (0.00)	10 (76.92)	3 (23.08)	13			0.404±0.136	
Normal weight	25 (3.96)	504 (79.87)	102 (16.17)	631	0.463±0.093		15 (2.84)	375 (70.89)	139 (26.28)	529			0.434±0.104	
Overweight	24 (3.15)	643 (84.49)	94 (12.35)	761	0.489±0.084Δ		26 (3.19)	629 (77.08)	161 (19.73)	816			0.470±0.090 Δ	
Obesity	6 (30.9)	161 (82.99)	27 (13.92)	194	0.487±0.089		12 (4.80)	181 (72.40)	57 (22.80)	250			0.467±0.089	
Statistics	χ2=7.63, P= 0.267				F=11.97, P<0.001		χ2=10.49, P= 0.105						F=18.34, P<0.001	
SMI grouping														
Low SMI	4 (1.83)	176 (80.73)	38 (17.43)	218	0.409±0.081		12 (2.76)	297 (68.43)	125 (28.80)		434		0.402±0.088	
Moderate SMI	18 (2.25)	663 (82.88)	119 (14.88)	800	0.471±0.086*		30 (3.54)	634 (74.85)	183 (21.61)		847		0.471±0.093*	
High SMI	35 (5.93)	485 (82.20)	70 (11.86)	590	0.513±0.081*#Δ		11 (3.36)	264 (80.73)	52 (15.90)		327		0.496±0.086*#Δ	
	χ2=19.31, P= 0.001				F=127.98, P<0.001		χ2=18.75, P= 0.001						F=120.36, P<0.001	
BFP grouping														
Low BFP	34 (5.81)	482 (82.39)	69 (11.79)	585	0.512±0.081†‡Δ		11 (3.51)	253 (80.83)	49 (15.65)		313		0.498±0.086†‡Δ	
Moderate BFP	18 (2.30)	650 (83.12)	114 (14.58)	782	0.474±0.085†		29 (3.54)	615 (75.00)	176 (21.46)		820		0.472±0.092†	
High BFP	5 (2.07)	192 (79.67)	44 (18.26)	241	0.409±0.084		13 (2.74)	327 68.84)	135 (28.42)		475		0.406±0.089	
	χ2=18.91, P= 0.001				F=132.28, P<0.001		χ2=18.77, P= 0.001						F=120.28, P<0.001	

BMI, body mass index; BMD, forearm bone mineral density; BFP, body fat percent (body fat/weight x100%); SMI, skeletal muscle mass index (total muscular mass/weight x100%).

*Compared with low SMI, P<0.05.

#Compared with moderate SMI, P<0.05.

†Compared with high BFP, P<0.05.

‡Compared with moderate BFP, P<0.05.

ΔHighest performance.

## Discussion

This was a retrospective study which examined the relationship between body composition and BMD from 56,462 individuals. Major findings from this study are that age, sex, BMI, iPTH, SMI, BFP, smoking, P and K+ were all significantly associated with osteoporosis, and that BMI, SMI, BFP and K+ were determined to be protective. Another notable finding is that blood levels of 25-(OH)D showed no statistically significant association with osteopenia or suspected osteoporosis. Of course, this may be related to the fact that we cannot rule out whether the elderly have taken vitamin D supplementation intervention.

Unsurprisingly, age and smoking were determined to be risk factors for osteopenia and osteoporosis, consistent with numerous previous studies (30, 31). Women were determined to be more at risk for osteoporosis, as expected based on a large body of clinical and experimental studies (14, 32). In this study, protective factors seemed to show a greater effect in women, likely due in part because the overall cohort included more men (57.58%) than women (42.42%). It is also notable that the percentage of women who shifted from normal BMD to suspected osteoporosis increased (*χ2 =* 357.43, *P*<0.001) in the baseline study.

The suspected osteoporosis group had the lowest BMI in the three groups, and multivariate analysis determined BMI to be a protective factor. At first glance, these findings would suggest that the higher the BMI, the lower the chance of developing osteoporosis. A deeper dive into these findings suggest a more complex interpretation of these results, however. Specifically, there was a significant positive association between BMD and BMI (*β*=0.1446, *P*<0.001). Furthermore, it is clear from longitudinal analysis of the 1,608 individuals who completed the ≥ 5-year follow-up that individuals who have an excessively low (i.e. underweight) or high BMI (i.e., obesity) are both more likely to develop osteoporosis. A large number of studies have confirmed that: first, people with an excessively low BMI tend to have malnutrition, whereas an updated America endocrine guideline in 2020 concluded that adequate protein intake helps to reduce bone loss (31) and that patients after bariatric surgery with major gastric resection have prevalent osteoporosis, also laterally reflecting the Association of malnutrition with osteoporosis or not just calcium and vitamin D supplementation (32). Second, groups with an excessively low BMI are often accompanied by a low SMI, and the mechanisms of osteoporosis with a low SMI are discussed later. And the association between obesity and osteoporosis, which is often explained by the fact that high BMI is positively associated with high BFP, and higher body fat rate and lower BMD, will be discussed later.

As further support of the association between low BMD and obesity, our findings showed a significant positive association between BFP the rate of suspected osteoporosis (*χ2 =* 524.72, *P*<0.001). Indeed, BMD and BFP had a significant negative association (*β*= -0.3839, *P*<0.001). This was not the case for SMI, however, as it was determined that the higher the SMI level, the lower the rate of suspected osteoporosis (*χ2 =* 538.85, *P*<0.001). There was also a significant positive association (*β*=0.3855, *P*<0.001) between BMD and SMI, suggesting a lower rate of osteoporosis among individuals with higher SMI or lower BFP. This result is consistent with most prior studies (16, 33, 34).

Our longitudinal analysis of the 1,608 individuals who received baseline and ≥ 5-year follow-up examinations showed that sex, smoking and drinking were not significant factors influencing new rates of suspected osteoporosis. All individuals experienced a similar increase in age across this sample, while simultaneously their BFP, BMI, FBG, HbA1c, Ca^2+^, K^+^ and 25-(OH)D levels also significantly increased from baseline. SMI, BMD, TC and LDL-C significantly decreased. These observed changes in FBG and HbA1c might be associated with aging. Since this study did not exclude individuals receiving lipid-lowering medication, the influence of such medication on the changes in TC and LDL-C which were observed cannot be ruled out. This study also did not exclude individuals receiving osteoporosis medications to which the increase of Ca^2+^, K^+^ and 25 (OH)D may have been connected. BFP, BMI and SMI are all modifiable factors and the change of SMI and BFP over the ≥ 5-year span may have affected BMI.

The longitudinal analysis further determined that at baseline, the group with a high SMI also had the highest BMD. BMD typically decreases with age, but individuals with a higher SMI have a greater bone mass and thus have a lower rate of osteoporosis. Similarly, individuals with a low BFP have the highest level of BMD. Taken together these data fully support the notion that lowering BFP and increasing SMI can help prevent osteoporosis (35, 36).

Theoretically, the positive association between SMI and BMD and the negative association between SMI and BFP can be attributed to three factors. The first and likely most important, according to some studies, is the mechanical forces between adjacent muscle and bone tissues. Given these forces, resistance exercise is a good way to increase SMI and BMD because this exercise causes these tissues to adapt in response to repetitive actions. The second important factor is the interaction and mutual promotion between the endocrine and paracrine actions of muscle and bone tissues. Skeletal muscle, particularly when contracting, can function as an endocrine organ and secrete myokines such as IGF-1 and irisin (37). Irisin secreted during exercise may play a role as a messenger in the muscle-fat-skeleton-brain axis, promoting energy consumption by fat cells, the differentiation of bone cells and suppressing the maturation of osteoclasts, thus influencing bone metabolism and enhancing bone density (38). The third factor is that increased BFP and enlarged fat cells cause sarcopenic obesity and promote chronic inflammation and insulin resistance. One study showed that apelin secreted by fat cells also regulates bone turnover and lowers BMD, increasing catabolism and leading to sarcopenia (39). Regardless of the underlying mechanisms, from a clinical perspective the most effective and appropriate strategy to prevent osteoporosis-related fractures is lifestyle modification (e.g., exercise and nutrition).

Since 2019, COVID-19 prevention measures such as travel bans, quarantines, and lockdowns, have had a seriously adverse effect on people’s lifestyle by reducing exercise, especially among the older adult population. Prolonged sedentary time is likely to increase BFP, lower SMI and reduce muscle force, which manifests as increased risk of falls and a possible surge in osteoporotic fractures. Thus, a greater attention to health management with respect to BMD is required in these times (40).

This study has several limitations, foremost of which are that it was a single-center study which used peripheral dual-energy X-ray absorptiometry for BMD measurement. These limitations were offset by the numerous merits of the study, including the large sample size which included a subset cohort with ≥ 5-year follow-up, use of consistent instrumentation and data harmonization due to the fact that the same medical staff performed every measurement in the entire cohort. Bioelectrical impedance is not a gold standard for evaluating body composition and have some disadvantages, and it is difficult to establish cause-and-effect relationships with cross-sectional design studies. Thus, our findings need further studies to confirm.

In conclusion, this study provides clear evidence that modifiable body composition indicators, including BMI, BFP and SMI, are all factors that significantly influence BMD. From a clinical perspective, these findings suggest that encouraging patients to adopt lifestyle measures to control BFP and increase SMI will help prevent osteoporosis.

## Data availability statement

The raw data supporting the conclusions of this article will be made available by the authors, without undue reservation.

## Ethics statement

The study protocol was approved by the Chinese People’s Liberation Army General hospital ethics committee and complied with the principles of the Declaration of Helsinki and its contemporary amendments. The patients/participants provided their written informed consent to participate in this study.

## Author contributions

YZ designed this study, acquired and analyzed the data. YL and ZH wrote the original drafts. YG wrote the review and prepared the tables. QZ reviewed and edited the manuscript. All authors read and approved the final manuscript. YL and ZH contributed equally as co-first authors. All authors contributed to the article and approved the submitted version.

## Conflict of interest

The authors declare that the research was conducted in the absence of any commercial or financial relationships that could be construed as a potential conflict of interest.

## Publisher’s note

All claims expressed in this article are solely those of the authors and do not necessarily represent those of their affiliated organizations, or those of the publisher, the editors and the reviewers. Any product that may be evaluated in this article, or claim that may be made by its manufacturer, is not guaranteed or endorsed by the publisher.
